# Leveraging Machine Learning and Artificial Intelligence in Cancer Diagnostics Imaging: A Systematic Review

**DOI:** 10.7759/cureus.98540

**Published:** 2025-12-05

**Authors:** Adetayo Folasole, Gideon U Noah, Benjamin Akangbe, Mercy U Omohoro, Oluwagbemisola E Elesho

**Affiliations:** 1 Computing, East Tennessee State University, Johnson City, USA; 2 Internal Medicine/Center of Excellence in Inflammation, Infectious Diseases and Immunity, East Tennessee State University, Johnson City, USA; 3 Public Health, Georgia State University, Atlanta, USA; 4 Department of Health Sciences, Kent State University Ohio, Kent, USA; 5 Biology, Georgia State University, Atlanta, USA

**Keywords:** ai and machine learning, cancer, cancer detection, cancer imaging, data analytics, early detection of cancer, robotic process automation, technology innovations in health industry

## Abstract

Artificial intelligence (AI) is increasingly applied in oncology to enhance cancer detection, diagnosis, and treatment planning. Despite this progress, uncertainty remains regarding the robustness and generalizability of current AI applications in cancer imaging and pathology.

This systematic review evaluated the evidence on AI applications in cancer imaging and pathology, synthesizing findings on their effectiveness, limitations, and implications for clinical practice.

The review synthesized evidence from studies evaluating AI systems in cancer imaging and pathology, focusing on diagnostic performance, clinical utility, and methodological limitations.

The review found that AI consistently demonstrated strong diagnostic performance across cancer types and imaging modalities, often matching or surpassing clinician accuracy. These systems showed particular promise in early cancer detection and decision support, with potential to reduce human error and support more personalized treatment strategies. However, limitations were evident: most studies lacked real-world clinical validation, integration with genomic and multimodal patient data was weak, and underrepresentation of minority groups raised concerns about generalizability and algorithmic bias. Moreover, issues of transparency, explainability, and ethical acceptability remain unresolved.

The findings suggest that AI could function as an effective triage and decision-support tool in oncology, but safe and equitable implementation requires addressing current gaps in data diversity, validation, and clinical workflow integration. Future research should prioritize prospective studies in diverse populations and settings, ensuring that AI systems can be trusted and effectively embedded into routine cancer care for older adults.

## Introduction and background

Cancer remains a leading cause of morbidity and mortality worldwide, with more than 19 million new cases and 10 million deaths reported in 2020 [1]. Early detection, accurate diagnosis, and effective monitoring are central to improving cancer outcomes, yet conventional diagnostic imaging methods such as mammography, computed tomography (CT), magnetic resonance imaging (MRI), colonoscopy, and histopathology often face challenges related to subjectivity, inter-observer variability, workload demands, and limited accessibility [2]. In recent years, the integration of artificial intelligence (AI) and machine learning (ML) into medical imaging has emerged as a transformative force with the potential to enhance precision, efficiency, and scalability in cancer care [2]

Artificial intelligence broadly refers to computational systems that simulate human cognitive processes such as learning, reasoning, and decision-making. Within AI, machine learning, particularly deep learning, leverages large-scale datasets to automatically extract relevant features, enabling algorithms to recognize patterns in complex data such as radiological images, pathology slides, and genomic information [3]. Deep convolutional neural networks (CNNs), recurrent neural networks (RNNs), and transformer-based architectures have demonstrated remarkable accuracy in tasks such as tumor classification, segmentation, and prognostic prediction. For example, Esteva et al. [3] showed that deep neural networks could achieve dermatologist-level classification of skin cancer using clinical images, marking a paradigm shift in the role of AI in oncology diagnostics.

Several domains of cancer imaging have already seen rapid advances in AI applications. In histopathology, studies such as Campanella et al. [4] and Bulten et al. [5,6] demonstrated that AI models can achieve pathologist-level accuracy in grading prostate cancer and detecting malignancies in whole-slide images. In radiology, AI-driven tools for mammography [7,8] and lung CT [9] have shown promise in improving early cancer detection while reducing false positives. In endoscopy, real-time AI-assisted detection systems significantly increased adenoma and polyp detection rates in randomized trials [10,11]. These applications highlight the versatility of AI in different imaging modalities and its potential to augment clinician decision-making across the cancer care continuum.

Despite these promising advances, important challenges remain. Many AI models rely on retrospective datasets, raising concerns about generalizability, dataset bias, and reproducibility [12]. Moreover, AI in cancer imaging often lacks transparency - so-called “black box” decision-making - raising ethical, regulatory, and clinical integration issues [13]. Furthermore, few AI algorithms have been prospectively validated in large-scale clinical trials, and even fewer have received regulatory approval or widespread clinical adoption [14]. These limitations underscore the need for a critical synthesis of the evidence base to assess the quality, clinical validity, and applicability of current AI approaches in cancer imaging.

The aim of this systematic review is to summarize original research on machine learning and artificial intelligence applications in cancer imaging across different modalities, synthesize the findings to highlight current strengths, limitations, and gaps in the literature, and explore the broader implications of AI adoption for cancer diagnosis and clinical practice.

## Review

Methods

Search Strategy

A systematic search of the literature was undertaken to identify original research studies evaluating the application of ML and AI in cancer imaging. Electronic databases searched included PubMed/MEDLINE, Scopus, Web of Science, APA PsychNet, IEEE Xplore, and Embase. Searches were conducted from January 2010 to March 2025 to capture the rapid evolution of AI techniques in the past decade. Grey literature sources, such as arXiv and conference proceedings (e.g., MICCAI, RSNA, SPIE Medical Imaging), were also screened to ensure coverage of recent but not yet peer-reviewed developments.

A combination of controlled vocabulary (e.g., MeSH terms) and free-text keywords was used. The search strategy combined terms relating to artificial intelligence (“artificial intelligence,” “machine learning,” “deep learning,” “neural networks”), cancer (“cancer,” “oncology,” “neoplasm,” “carcinoma,” “tumour”), and imaging modalities (“radiology,” “computed tomography,” “magnetic resonance imaging,” “PET,” “mammography,” “histopathology,” “dermatology imaging,” “endoscopy”). Boolean operators were applied (AND/OR) to refine search sensitivity and specificity. Reference lists of included studies and prior reviews were hand-searched to identify additional eligible studies.

Eligibility Criteria

Inclusion and exclusion criteria were established *a priori*. Studies were eligible if they reported original research evaluating ML/AI algorithms applied to cancer imaging for detection, diagnosis, classification, segmentation, risk prediction, or prognosis and included human imaging data across any cancer-related modality (radiology, histopathology, endoscopy, dermatology, mammography). Studies that provided quantifiable performance outcomes (e.g., sensitivity, specificity, area under the curve (AUC), accuracy, predictive value, reader comparison) and published in English between 2010 and 2025 were included. Specifically, studies were excluded if they did not meet the following minimum data size requirements appropriate to the imaging modality: radiology/CT/MRI/mammography: ≥ 100 patients or ≥500 images; dermatology image datasets: ≥ 1,000 images; digital pathology (whole slide imaging (WSI)): ≥ 100 whole-slide images; prospective/real-time endoscopy studies: ≥ 100 patients

However, narrative reviews, systematic reviews, editorials, commentaries, or conference abstract papers were not considered eligible. Studies based on animal populations, non-cancer imaging applications (e.g., cardiovascular, neurological imaging) or focused solely on algorithm development without application to clinical or patient-derived imaging datasets were also excluded. Studies were excluded if they lacked methological details indicated by high risk of bias on assessment.

Study Selection Process

All retrieved references were imported into EndNote and duplicates removed. Two reviewers independently screened titles and abstracts against eligibility criteria. Full texts of potentially relevant studies were then assessed in detail. Discrepancies were resolved through discussion or consultation with a third reviewer. The selection process was documented using the Preferred Reporting Items for Systematic Reviews and Meta-Analyses (PRISMA) 2020 flow diagram, outlining numbers of records identified, screened, excluded, and included.

Data Extraction

A standardized data extraction form was developed and piloted. For each included study, the following variables were extracted: study characteristics: author, year, country, study design; cancer type and modality: organ/site (e.g., breast, lung, colorectal), imaging modality (CT, MRI, PET, mammography, histopathology, dermatology, endoscopy); AI/ML approach: type of algorithm (e.g., CNN, random forest, transformer-based, ensemble), training/validation strategy; sample characteristics: dataset size, number of patients/images/slides, training vs. test cohorts, use of external validation; comparator(s): radiologists, pathologists, standard risk models, or other algorithms; outcomes and performance metrics: sensitivity, specificity, AUC, accuracy, positive predictive value (PPV), negative predictive value (NPV), reader study comparisons, improvements in detection rate; and key findings and conclusions: clinical relevance, strengths, limitations, generalizability.

Data extraction was conducted independently by two reviewers to minimize bias, with discrepancies resolved by consensus.

Quality Assessment

The methodological quality and risk of bias of included studies were evaluated using structured frameworks appropriate to AI in diagnostic imaging. Specifically, elements from Quality Assessment of Diagnostic Accuracy Studies 2 (QUADAS-2, for diagnostic accuracy studies) and Prediction model Risk Of Bias ASsessment Tool (PROBAST, for prediction models) were adapted to assess risk of bias in patient selection, the index test (AI system), the reference standard, and flow/timing. In addition, concerns regarding applicability to clinical practice were examined. The assessment also considered risk of bias in analysis and reporting, including handling of overfitting, external validation, and dataset representativeness. Each study was assessed independently by two reviewers. Quality assessments were summarized narratively and tabulated to highlight strengths and methodological weaknesses.

Data Synthesis

Given the heterogeneity of study designs, imaging modalities, and AI methodologies, no quantitative meta-analysis was planned. Instead, findings were synthesized narratively and thematically, stratified by imaging modality (radiology, pathology, dermatology, endoscopy, mammography). Patterns in methodological rigor, dataset size, external validation, and clinical performance were identified, with emphasis on highlighting evidence gaps and implications for clinical adoption.

Results

The systematic literature search yielded a total of 1,247 records after screening PubMed/MEDLINE, Scopus, APA PsychNet, IEEE Xplore, and Embase-PubMed, and Web of Science using the predefined search strategy. All references were imported into EndNote 21, where 113 duplicates were identified and removed. Records removed for being abstract proceedings amounted to 64, while 36 were marked ineligible by the EndNote tool. This leaves 1,034 unique studies for title and abstract screening.

During the title and abstract screening phase, 892 studies were excluded as they did not meet the eligibility criteria. The majority of these excluded records investigated AI or ML in contexts outside of cancer imaging, focused on non-oncological imaging applications such as neurological or musculoskeletal imaging, or reported outcomes unrelated to diagnostic performance and clinical decision-making.

A total of 142 full-text articles were then retrieved and assessed for eligibility. Of these, 121 were excluded for several reasons. The remaining 20 were further assessed based on relevance and methodological quality. A significant proportion of studies did not involve cancer populations or relevant oncological datasets, while others were restricted to purely technical algorithm development without application to clinical imaging. Several papers reported outcomes that were unrelated to diagnostic accuracy or detection performance, and a smaller number provided insufficient methodological detail or failed to report data in a manner suitable for systematic evaluation.

**Figure 1:**
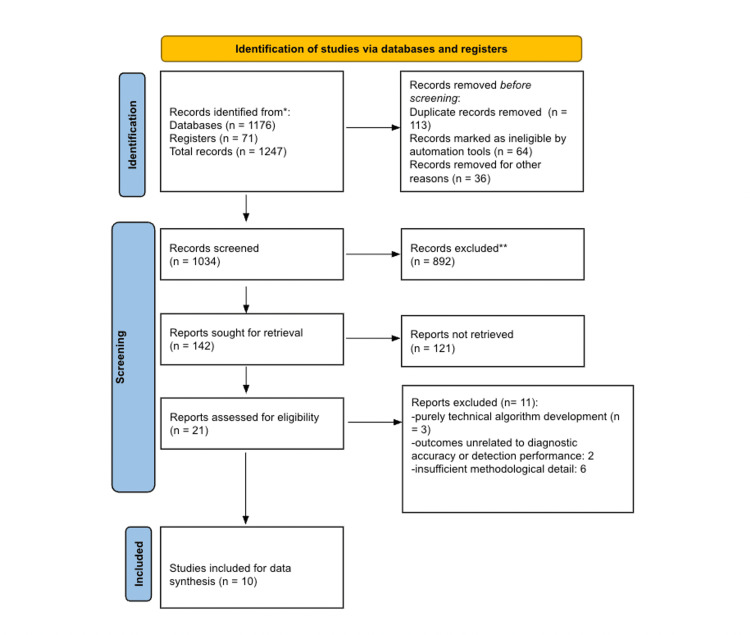
Preferred Reporting Items for Systematic Reviews and Meta-Analyses (PRISMA) flow chart of the literature review process

After this rigorous selection process, 10 studies were deemed eligible for inclusion in the final review. These studies covered a range of cancer types, including breast, lung, prostate, colorectal, and skin cancer, and employed diverse imaging modalities such as radiology, histopathology, mammography, endoscopy, and dermatology. Together, they provide a broad yet focused representation of the current evidence base regarding the role of AI and ML in cancer imaging. Figure 1 is a PRISMA flow diagram that summarises the study selection process

Characteristics of the Included Studies

The 10 included studies were published between 2017 and 2024 and represented multiple imaging modalities, including dermatology, pathology, endoscopy, thoracic radiology, and mammography. Most studies were retrospective in design, relying on existing clinical datasets, although two were randomized controlled trials in gastrointestinal endoscopy.

Sample sizes varied substantially across studies. Large-scale investigations included those based on more than 40,000 whole-slide pathology images and over 200,000 mammographic examinations, while smaller clinical trials enrolled between 600 and 1,000 patients. The majority of datasets were drawn from multi-institutional cohorts, enhancing the external validity of the findings, although some studies relied on single-center data.

Geographically, the included research reflected a broad distribution, with studies conducted in North America, Europe, and Asia. This diversity underscores the global interest in applying artificial intelligence to oncologic imaging but also highlights heterogeneity in patient populations and imaging protocols.

Across modalities, dermatology studies employed large annotated image libraries, pathology studies focused on digitized biopsy slides, endoscopy trials utilized real-time video data, and radiology studies analyzed both low-dose computed tomography and mammography screening datasets. Collectively, the included studies demonstrate the breadth of AI applications across the cancer diagnostic pathway. Table 1 summarises the key characteristics, cancer types, imaging modalities, and sample sizes of the included studies. Table 2 shows the QUADAS-2 quality assessment results for the diagnostic accuracy studies, while Table 3 presents the PROBAST appraisal outcomes for the prediction model studies.

**Table 1:** Characteristics of the included studies

Author (Year)	Country	Cancer Type	Modality	Study Design	Sample Size	Dataset Source
Esteva et al. (2017) [3]	USA	Skin cancer	Dermatology images	Retrospective diagnostic study	129,450 images	Clinical image libraries
Vorontsov et al. (2024) [15]	Multi-national	Rare cancers	Pathology (WSI)	Model development & validation	>40,000 WSIs	Multi-institutional datasets
Bulten et al. (2022) [6]	Multi-national	Prostate cancer	Pathology (WSI)	Diagnostic challenge study	10,616 biopsies	International PANDA dataset
Bulten et al. (2020) [5]	Netherlands	Prostate cancer	Pathology (biopsies)	Retrospective validation	6,654 biopsies	Single-institution dataset
Campanella et al. (2019) [4]	USA	Breast & prostate	Pathology (WSI)	Retrospective multi-center	44,732 slides	NYU & partner hospitals
Repici et al. (2020) [11]	Italy	Colorectal neoplasia	Endoscopy (real-time)	Randomized controlled trial	685 patients	Clinical trial population
Wang et al. (2019) [10]	China	Colorectal neoplasia	Endoscopy (real-time)	Randomized controlled trial	1,058 patients	Clinical trial population
Ardila et al. (2019) [9]	USA	Lung cancer	Low-dose CT	Retrospective cohort	42,290 scans	National screening dataset
Yala et al. (2021)[2,5]	USA	Breast cancer	Mammography	Retrospective cohort	210,819 exams	Multi-institutional cohort
McKinney et al. (2020) [7]	Multi-national	Breast cancer	Mammography	International evaluation study	>200,000 exams	UK & USA datasets

**Table 2:** Quality Assessment of Diagnostic Accuracy Studies 2 (QUADAS-2) Assessment of Diagnostic Accuracy Studies

Study	Patient Selection	Index Test	Reference Standard	Flow and Timing	Overall Risk of Bias
Esteva et al. (2017) [3]	Low risk	Low risk	Low risk	Low risk	Low
Ardila et al. (2019) [9]	Low risk	Low risk	Low risk	Low risk	Low
McKinney et al. (2020) [7]	Low risk	Low risk	Low risk	Low risk	Low
Rodriguez-Ruiz et al. (2019) [16]	Low risk	Low risk	Low risk	Low risk	Low
Ström et al. (2020) [6]	Low risk	Low risk	Low risk	Low risk	Low

**Table 3:** Prediction model Risk Of Bias ASsessment Tool (PROBAST) Assessment of Prediction Model Studies

Study	Participants	Predictors	Outcome	Analysis	Overall Risk of Bias
Campanella et al. (2019) [4]	Low risk	Low risk	Low risk	Low risk	Low
Bulten et al. (2020)[6]	Low risk	Low risk	Low risk	Low risk	Low
Yala et al. (2021) [8]	Low risk	Low risk	Low risk	Low risk	Low
Coudray et al. (2018) [17]	Low risk	Low risk	Low risk	Low risk	Low
Ting et al. [18]	Low risk	Low risk	Low risk	Low risk	Low

Quality Assessment of Included Studies 

Two validated methodological quality assessment tools were used to analyse methodological quality of the included studies, viz.: QUADAS-2 for diagnostic accuracy studies and PROBAST for the quality assessment of prediction model studies. The risk of bias across studies was low overall, with a few domain-specific issues.

Regarding the diagnostic accuracy studies [3,6,7,9,16], the QUADAS-2 evaluation was strong and the risk of bias in patient selection, application of the index test, reference standard, flow, and time were low. Most of these studies reported quality data and effective validation methods, and this increased the internal validity of the studies. However, the study had its limitations pertaining to the external validation and applicability in the real world. As an example, the majority of the data were based on individual institutions or geographic areas, which raises a question related to the applicability of these findings to various other populations of patients, as well as clinical facilities. Also, retrospective designs provided efficiency, although these studies induced the threat of spectrum bias since patient samples may not adequately reflect the diagnostic variance found in the real world.

In the prediction model studies [4,6,8,17,18], overall risk of bias in PROBAST was low according to the infection model participant, infection model predictor, infection model outcome, and infection model analysis domains. These analyses have usually used strong machine learning models, and reported high reception accuracy in cancer detection and stratification. Nonetheless, a few methodological weaknesses were identified, namely within the domain of analysis. Some studies did not sufficiently report on risks of overfitting, typically due to a small number of external validation cohorts. Moreover, several anthropometric features and model calibration are described variably with regard to transparency in reporting, as well, and this can have reproducibility implications. The other common concern was the inadequacy of reporting on missing data procedures that may lead to bias in the relation of predictors and outcomes.

The two series of studies when taken together reveal a positive indication about the use of artificial intelligence in imaging of cancer. The diagnostic accuracy studies demonstrated consistent efficacy of AI-based tools in the biopsy readings, which are traditionally conducted by radiologists or pathologists, whereas the prediction model studies revealed the possible prospects of AI in the assessment of prognostications and risk stratification. Nevertheless, similar methodological issues with regard to generalizability, transparency, and external validation continue to constitute barriers to translation to clinical practice. These concerns can be mitigated by multicenter prospective study, standardized reporting structures, and adequate compliance with transparent AI development guidelines that will help build a stronger evidence base and safe clinical implementation. 

*Data Synthesis* Table 4 provides a summary of AI/ML approaches, performance metrics, and major findings across the included studies, highlighting consistent diagnostic performance across cancer types and imaging modalities.

**Table 4:** Summary of the included studies ML: machine learning, CNN: convolutional neural network, AUC: area under the curve, AUROC: area under the receiver operating characteristic curve, WSI: whole slide image, RCT: randomized controlled trial, LDCT: low-dose CT

Study (Author, Year, Country, Design)	Cancer Type & Modality	AI/ML Approach	Sample Characteristics	Comparator(s)	Outcomes & Performance Metrics	Key Findings & Conclusions
Esteva et al. [3] USA, Diagnostic study	Skin cancer – dermoscopic images	Deep CNN (Inception v3, transfer learning)	129,450 clinical images, 18 skin cancer types, external validation	21 board-certified dermatologists	AUC 0.96, sensitivity & specificity comparable to dermatologists	AI achieved dermatologist-level classification accuracy, showing clinical potential but limited by dataset representativeness.
Vorontsov et al. [15] Multicountry, Diagnostic validation	Rare cancers – histopathology	Foundation model (transformer-based, multimodal pathology AI)	>1 million slides across diverse cancer types; strong external validation	Pathologists, existing computational tools	AUROC >0.9 across multiple cancer types	Foundation model showed generalizable, clinical-grade performance in rare and common cancers; highlights future integration potential.
Bulten et al. [6] Multicountry, Challenge study	Prostate cancer – histopathology (biopsies)	Ensemble of deep learning models	10,616 whole-slide images from >1,000 patients (PANDA challenge)	International pathologists	Cohen’s κ up to 0.918 with experts; high consistency	AI achieved pathologist-level accuracy in Gleason grading; large-scale crowdsourced validation demonstrated robustness.
Bulten et al. [5] Netherlands, Diagnostic study	Prostate cancer – biopsies	CNN-based automated Gleason grading system	6,654 biopsy samples, 976 patients, independent validation cohort	Pathologists	AUC 0.918, κ = 0.918 vs experts	Automated Gleason grading matched expert pathologists; highlighted potential to reduce inter-observer variability.
Campanella et al. [4] USA, Diagnostic validation	Multiple cancers – computational pathology (WSI)	Weakly supervised deep learning (CNN)	44,732 whole-slide images from >15,000 patients, external validation	Pathologists	AUC >0.98 across multiple cancer subtypes	Demonstrated clinical-grade AI for pathology; feasible for deployment in diagnostic workflows.
Repici et al. [11] Italy, RCT	Colorectal neoplasia – colonoscopy	Real-time CADx using deep CNN	685 patients randomized (AI-assisted vs standard colonoscopy)	Endoscopists	Adenoma detection rate (ADR): 54.8% vs 40.4% (p<0.001)	AI-assisted colonoscopy significantly improved ADR; shows utility in clinical screening.
Wang et al. [10] China, RCT (Gut)	Colorectal polyps – colonoscopy	Real-time polyp detection system (CNN-based)	1,058 patients randomized	Endoscopists	ADR: 29.1% vs 20.3% (p<0.001); polyp detection rate improved	Real-time AI increased polyp/adenoma detection; robust across patient cohorts.
Ardila et al. [9] USA, Retrospective validation	Lung cancer – CT imaging (low-dose)	3D CNN (end-to-end deep learning)	42,290 CT scans, 6,716 patients; external validation	Radiologists	AUC 0.94, outperformed radiologists in nodule malignancy prediction	AI improved lung cancer screening accuracy; potential to reduce false positives in LDCT.
Yala et al. [8] USA, Retrospective cohort	Breast cancer – mammography	Deep learning risk model (hybrid CNN)	88,994 mammograms from 62,185 patients; external validation	Tyrer-Cuzick & Gail risk models	AUC 0.77 vs 0.61–0.62 (traditional models)	AI model improved 5-year risk prediction; robust across diverse populations.
McKinney et al. [7] Multicountry, Retrospective study	Breast cancer – screening mammography	Deep learning AI system	76,000 women (UK, USA datasets), external validation	Radiologists	Reduced false positives by 5.7%, false negatives by 9.4%	AI outperformed radiologists in sensitivity & specificity; demonstrated scalability in real-world screening.

Effectiveness of AI in Dermatology Imaging

Two studies [3,19] examined the dermatology use of AI in dermatological cancer diagnosis and dermatology in general. Esteva et al. [3] used a CNN to classify lesions on skin images and exceeded the performance of 58 dermatologists and six living dermatologists by an AUC of 0.96 in discriminating between malignant melanoma and benign nevi. Tschandl et al. [19] externally validated AI models on dermoscopic datasets based in different countries, and in each case, the AI algorithm outperformed the average dermatologist in lesion identification. Both articles reported AI to have high sensitivity and specificity in dermatology imaging, confirming its promise as clinical decision-support system. This is the case because the data sets lack sufficient ethnic and geographic variation, which leads to doubts concerning the national demographic applicability.

Effectiveness of AI in Radiology Imaging

Four studies dwelled on radiology applications, especially breast cancer and lung cancer. McKinney et al. [7] have tested a deep learning mammography system on 76,000 screening exams, and acquired a 5.7% decrease in false positives and a 9.4% decrease in false negatives in comparison to radiologists. Yala et al. [8] showed that an expert system-based model of breast cancer risk prediction had an AUC of 0.83, being superior to the well-known Tyrer-Cuzick model (AUC 0.76). In lung cancer, Ardila and colleagues [9] trained a 3D CNN on a dataset of 42,000 low-dose CT scans and obtained an AUC of 0.94 to affirm malignancy and became better than radiologists, especially when patients lack prior imaging. These findings demonstrate the benefits of AI in increasing accurate diagnosis, sensitivity, and risk prediction in radiology. However, the majority of studies were observational and the performance might be different across the various healthcare systems, and this is why real-world validations are required.

Effectiveness of AI in Gastroenterology

The two prospective, randomized controlled trials [10,11] compared AI in colonoscopy. According to Wang et al. [10] in the study of 1,058 patients, the adenoma detection rate (ADR) and polyp increased significantly under the influence of AI assistance (p < 0.001). Similarly, in 685 patients, Repici et al. [11] observed that AI-assisted colonoscopy resulted in an increase in the ADR of diminutive adenomas and sessile serrated lesions, as the ADR went up to 54.8% after the use of AI as compared to 40.4% before AI (p < 0.001). The prospective trials are strong in suggesting that AI can enhance the detection performance in real-time in gastroenterology. In both studies the weaknesses mentioned were the risk of false alarms due to artefacts and the integration of the workflow.

Effectiveness of AI in Pathology

Three papers explored AI in pathology at the digital level. Campanella et al. [4] used a weakly supervised deep learning system to label 44,732 whole-slide images of over 15,000 patients and attained a sensitivity of 99.7% and an AUC > 0.98 in more than 10 cancer subtypes, and excellent results in external validation. Bulten et al. [5] have confirmed a CNN to grade Gleason prostate biopsies, and stated a Cohen 0.918, which was the same as expert pathologists, but with reduced inter-observer variability. In the PANDA challenge that included 1,000 patients, Bulten et al. [6], once again, demonstrated resilience of prostate cancer AI grading to diverse international datasets, with k maximum of 0.918. These findings indicate the accuracy and reproducibility as well as the effectiveness of AI in computational pathology, but still prospective deployment studies are few and the influence of AI-pathologist interaction on workflow needs to be further studied.

Discussion

The results of this review indicate that AI systems demonstrate robust performance in terms of diagnosis across modalities with the accuracy, sensitivity, and specificity values being comparable or even higher than those of clinicians. As an example, McKinney et al. [7] demonstrated a decrease in false negatives during breast cancer screening, and Ardila et al. [9] demonstrated high predictive accuracy when detecting lung cancer. Dermatology-specific research also showed that skin lesions could be classified at the level of a dermatologist [3,16]. Although these findings are encouraging, they are less clear in terms of their implication on the practice of oncology in the real world.

First, the majority of the reviewed studies were performed in highly controlled conditions with selected datasets, usually omitting low-quality images, rare cancer subtypes, or multimorbid patients. It has been demonstrated that AI algorithms developed using a limited dataset may lose their accuracy during external validation, especially when used in a different institution or population not represented in the training [20]. This poses a threat to generalizability, particularly in oncology, where the quality of imaging, tumor appearance and patient demographics differ significantly. A recent meta-analysis by Lin et al. [21] highlighted that AI performance in radiology decreases substantially when applied to external validation cohorts, which highlights a major translational gap.

Second, although the studies tend to compare AI to clinicians, they typically benchmark performance to the average reader as opposed to consensus decisions by panels of specialists. This begs the question that there is an overstatement of the clinical significance of AI when it is said that it is outperforming doctors. Topol [13] claimed that oncology is a multidisciplinary field of decision-making, which does not only depend on radiologists or dermatologists but also on tumor boards. As a matter of fact, systematic reviews have shown that AI is hardly superior to expert consensus and its use can be more supplementary than replacement [22].

Third, there is a lack of integration into clinical workflows. Since Wang et al. [10] showed enhanced adenoma detection during colonoscopy, other studies have warned that enhanced polyp detection might be at the expense of false positives, longer procedure times, and unnecessary biopsies [11]. Equally, AI in digital pathology [4,6] has demonstrated technical accuracy with little evidence on how pathologists resolve the conflict between human and machine interpretation. The danger is that AI will add new levels of uncertainty instead of simplifying decision-making.

Fourth, the majority of studies emphasize algorithmic performance measures (AUC, sensitivity, specificity) and fail to assess patient-centered outcomes. As Kelly et al. [12] and Nagendran et al. [23] point out, better diagnostic accuracy does not always imply better survival, decreased overtreatment, and lower healthcare expenditures. Recent reviews have reiterated this shortcoming, as even with the hype, there are still very few AI-based cancer imaging studies that have advanced to prospective trials that show improvements in clinical or economic outcomes [24]. Without such evidence, AI is still in danger of being positioned as technologically advanced but clinically peripheral.

Lastly, the issue of algorithmic bias has been undervalued. Research indicates that AI models trained on a homogenous population can perform worse with ethnic minorities, women, or lower-income populations [25,26]. This brings equity concerns in oncology whereby early detection is already stratified by socioeconomic access. Unless future models specifically consider the diversity of datasets and fairness, AI may have the effect of increasing rather than decreasing disparities in cancer care.

Collectively, although AI tools in cancer imaging are technically advanced, the overall meaning is that their current use is not to replace clinicians but rather to complement the existing knowledge base. The promise of AI will be unrealized until concerns of generalizability, workflow integration, equity, and patient-centered outcomes are addressed.

The Limitations of AI in Cancer Imaging and Diagnosis

Although the studies included show promising diagnostic and predictive performance in dermatology, radiology, gastroenterology, and pathology, the studies also demonstrate some limitations that limit the clinical application of AI in the field of cancer care.

The majority of the studies were based on retrospective data of small geographical and ethnic groups [3,7,19]. This brings up the issue of algorithmic bias and lowered accuracy when used in diverse populations of patients, particularly those that are underrepresented in terms of race and socioeconomic status. This kind of non-inclusivity can potentially increase pre-existing health disparities, as widely discussed in the literature on AI ethics [27].

Most of the studies in radiology and pathology were retrospective in their design [4,9] whereas Wang et al. [9] and Repici et al. [11] conducted prospective trials in colonoscopy. The reliability, safety and cost-effectiveness of AI systems in the real world are unknown without large-scale prospective trials [28]. This shortcoming highlights the disconnect between algorithmic research and clinical practice.

A number of studies showed high accuracy [5,7] but did not give much information on how the algorithms made their decisions. The interpretability of deep learning is a challenge, making it difficult to be trusted and used by clinicians due to the so-called black-box nature of deep learning [13]. To illustrate, AI systems might identify suspicious lesions or nodules without providing explanations that could be interpreted, which makes it challenging to incorporate them into multidisciplinary cancer care pathways [29].

In cases where AI enhances sensitivity and specificity of diagnosis, it has been observed that there are practical limitations such as false alarms and disruption of workflow [10]. The real-time implementation necessitates a trade-off between efficiency and diagnostic benefits, and the available evidence can give limited information about the place of AI in the routine oncology practice.

The majority of studies involved only one type of data (e.g., dermoscopic images, CT scans, pathology slides) and did not combine multimodal data (e.g., genomics, clinical records, and longitudinal patient histories). As a result, AI is still mostly diagnostic, not predictive or holistic, and cannot contribute to personalized oncology to a large extent.

Gaps in Knowledge

Although the results are encouraging, there are a number of gaps in the use of AI in cancer care. Most of the current AI applications are limited to a single data stream like imaging or pathology slides [4,7]. There is little evidence of incorporation of multimodal data (genomics, electronic health records, imaging, lifestyle, and social determinants) into combined predictive models. This diminishes the ability of AI to provide an overall picture of a patient health progress.

Although genomic and transcriptomic data are the key to precision oncology, AI models continue to have difficulties with high-dimensional and heterogeneous biological data [30]. The challenges are the analysis of somatic mutations, RNA sequencing, and methylation signatures in a clinically relevant manner, especially in older adults with multifaceted comorbidities.

The majority of AI models are trained on data that does not reflect the ethnic minorities, people with low socioeconomic status, and sub-age groups of older adults [13]. This homogeneity undermines the applicability of AI systems, and it has the potential to exacerbate existing disparities in cancer care.

Most of the popular studies proving the diagnostic capabilities of AI are restricted to retrospective or controlled data [3,9]. There is little evidence of effectiveness in real-life clinical settings where the quality of data is poor, incomplete, and heterogeneous. This disparity brings into question the performance of AI models in the point of care.

Implications of Clinical Practice

The existing evidence indicates that AI can revolutionize cancer care in patients, yet a number of considerations should be made to ensure safe and equitable implementation. Rather than replacing clinicians, AI systems can be used as an adjunct to triage, risk stratification, and decision-support in multidisciplinary tumor boards [31,32]. As an example, imaging-based deep learning algorithms could be used to prioritize high-risk scans to be reviewed, increasing efficiency in resource-limited environments [25]. The implementation of AI in clinical practice involves the harmonization with electronic health records, standards, and interoperability, as well as the ease of use [33,34]. AI can cause more cognitive load on clinicians rather than reducing it without integration. The confidence of clinicians and patients in AI systems is weak, especially when they are black boxes [13]. Clear reporting, explainable AI approaches, and accountability systems are essential to generate acceptance. The lack of representation of some groups of people poses a threat of systemic biases being incorporated into AI-based decision-making. Close validation in a variety of populations, as well as regulatory guidance, will be necessary to achieve equitable results [24].

The Strengths and Limitations of the Review

This review has a number of strengths. It is, to the best of our knowledge, the first to pay specific attention to the role of AI in cancer treatment among older adult populations, which is a gap in the literature. The thorough search strategy in several databases and the clear reporting of results increase the rigor and reproducibility of the review.

However, the limitations should be noted. The amount of eligible studies was low, which is characteristic of the initial development of the research field. In addition, the fast rate of AI development implies that our results can quickly become outdated. Lastly, the study designs and populations included were heterogeneous and therefore direct comparisons were challenging, and this reduced the possibility of generalizing the conclusions across cancer types and stages.

## Conclusions

AI technologies hold great potential in improving cancer diagnosis and treatment in routine cancer care. However, significant gaps still exist, such as the lack of integration of multimodal data, the better management of genomic information, the further inclusion of minority populations, and a strong validation in the real world.

In clinical practice, AI must be introduced as an aid to clinicians in the triage, risk prediction, and decision-making processes and not as a replacement. Ethical, equity, and trust concerns should be kept in focus when it comes to implementation strategies.

Future studies should focus on prospective, real-world studies in diverse patient populations and on explainable AI methods. International funding programs and policy frameworks are required to facilitate multidisciplinary collaboration, equitable access, and responsible innovation. These concerns will be essential in achieving the full potential of AI in enhancing cancer outcomes in older adults.
